# Low prevalence of myopia among school children in rural China

**DOI:** 10.1186/s12886-018-0808-0

**Published:** 2018-06-11

**Authors:** Chen-Wei Pan, Rong-Kun Wu, Jun Li, Hua Zhong

**Affiliations:** 1 School of Public Health, Medical College of Soochow University, Suzhou, China; 2 Department of Ophthalmology, the Second People’s Hospital of Yunnan Province, Kunming, China; 3 Department of Ophthalmology, the First Affiliated Hospital of Kunming Medical University, 295 Xi Chang Road, Kunming, 650032 China

**Keywords:** Myopia, Chinese, Prevalence, Risk factors

## Abstract

**Background:**

We aim to assess the prevalence of myopia in Chinese school children with low educational pressure and explore which factors could explain the differences in prevalence between generations.

**Methods:**

A school-based epidemiologic study including 2432 grade 1 and 2346 grade 7 students was conducted from 2016 in rural areas of China. Each participant’s refractive status was measured before and after cycloplegia using an autorefractor and axial length (AL) was measured using an IOL Master. The questionnaires were completed by the parents or legal guardians of the children to collect detailed information regarding risk factors. Myopia was defined as spherical equivalent less than − 0.50D.

**Results:**

Grade 7 students had a higher prevalence of myopia (29.4% vs. 2.4%; *P* < 0.001) and high myopia (0.4% vs. 0.1%; *P* < 0.001) compared with grade 1 students. Grade 7 students also had longer ALs (23.50 mm vs. 23.37 mm; *p* = 0.004) after adjusting for the effect of gender, height and other myopia-related risk factors. Adjustment for time spent on reading and writing after school per day led to a reduction in the excess prevalence of myopia in grade 7 students by 15.1%. In addition, adjustment for time outdoors reduced the excess prevalence of myopia in grade 7 students by 33.4%.

**Conclusions:**

We reported a relatively lower prevalence of myopia in school students in rural China, suggesting that Chinese may not have a genetic predisposition to myopia and environmental factors may play a major role in the development of school myopia in Chinese children.

## Background

Myopia is a worldwide health concern and its burden has increased rapidly in recent years [1–3]. Several studies have revealed significant ethnic variations in the prevalence of myopia and it is always reported that myopia is more prevalent in Chinese compared with other ethnic groups [4–7]. Thus, research regarding the epidemiology of myopia in Chinese population is a major interest to global myopia investigators.

The reasons underlying the observed disparities in myopia prevalence between Chinese and other ethnic groups remain unclear [8]. Ethnicity is a proxy measure for the differences in both genetic biomarkers and environmental exposures such as the intensity of schooling, near work activities, time outdoors, and other lifestyle factors [9, 10]. In the past decades, genetic studies including linkage analyses, candidate gene and genome-wide association studies, and next-generation sequencing studies have substantiated the gene mapping for myopia in Chinese [11–16]. One may hypothesize that Chinese may have a genetic predisposition to myopia. An alternative hypothesis is that environmental factors play a major role as Chinese culture emphasizes on very early educational achievements and passing examinations. Therefore, Chinese children may have devoted more time to rigorous learning and less time in outdoor play in their early years of life compared their counterparts of other ethnicities.

Comparison of the prevalence of myopia between those who have just started schooling and those who have completed several years of education living in the same region may help to elucidate how environmental exposures could explain the differences in myopia prevalence between generations. In this study, we reported the prevalence of myopia in two samples of Chinese school children (grade 1 students and grade 7 students) in a rural county in China, where educational pressure such as schooling systems and parental expectation on academic achievements is less intensive than many of the other Chinese communities. We also explored which factors could explain the differences in prevalence between the two samples.

## Methods

### Study population

The Mojiang Myopia Progression Study is a school-based cohort study in rural China. The study protocol has been described elsewhere [17–19]. The study included two samples: elementary school grade 1 students and middle school grade 7 students. Elementary school grade 1 students would be followed until they entered middle schools and middle school grade 7 students would be followed until they entered high schools. Such a study design would facilitate the follow up of the samples and possibly reduce the loss-to-follow-up rate considering the current Chinese schooling system. The baseline survey was conducted in 2016 and all the participants were followed annually. Mojiang is a small county with a population of 0.36 million in Southwestern China. The compulsory schooling system is well executed in Mojiang and school-based samples are highly representative of the local population.

We invited all the grade 1 students from elementary schools and grade 7 students from middle schools in Mojiang to participate in this study. The students roster was provided by each school’s principal to ascertain the eligibility of the study participants. Students who had been living in Mojiang for at least 1 year and would live there for at least 4 years were included. Before the study, school principals had contacted the parents asking about their plan. A cell phone message was sent to the parents to invite them to participate in the study. Telephone calls were made if the parents did not respond to the cell phone message. In the end of the study, a total of 2432 (response rate: 90.2%) grade 1 students and 2346 (response rate: 93.5%) grade 7 students participated in the baseline survey.

The Mojiang Myopia Progression Study was approved by the Institutional Review Board of Kunming Medical University. Written informed consents was obtained from at least one parent or legal guardian of each participant.

### Refractive error and ocular biometry measurement

Refractive error was measured after cycloplegia using an autorefractor (RM-8000; Topcon Corp., Tokyo, Japan). Myopia was defined as spherical equivalent (SE) less than − 0.50 diopter (D). Ocular biometric parameters such as axial length (AL) were measure using an IOL Master (Carl Zeiss Meditec AG, Jena, Germany).

### Questionnaires

The World Health Organization (WHO) myopia risk factor questionnaire was used in this study [20–23]. The questionnaires were completed by the parents or legal guardians of the children. We collected detailed information regarding socioeconomic status, parental education, parental history of myopia, medical history, time spent on reading and writing, time spent on watching TV, time spent on playing computers and outdoor activities.

### Statistical analysis

Data were analyzed using a commercial statistical software (Statistical Package for Social Science, SPSS V16.0; SPSS Inc., Chicago, IL). As the correlation of SEs (*r* = 0.90) and ALs (*r* = 0.98) between two eyes was high (*r* = 0.90) and the results of analysis in both eyes were similar, only data for the right eye are presented in this paper. Distribution for refractive error and ocular biometric parameters was tested for normality with the Kolmogorov-Smirnov test and were considered significantly different from normal when *P* value was less than 0.05. Prevalence were estimated with mixed models being fitted to adjust for clustering within schools. Where cluster effects were not significant, *t*-tests and normal linear regression were used. All confidence intervals (CIs) are 95%.

To evaluate the extent that myopia-related lifestyle risk factors may explain the excess prevalence of myopia in grade 7 compared with grade 1 students, we estimated the percentage reduction in odds associated with adjustment for myopia-related lifestyle risk factors according to the following formula: (Ra-Rb)/(Ra-1) x 100, where Ra is the odds ratio (OR) of myopia in grade 7 compared with grade 1 students, adjusted for gender only (reference model), and Rb is the OR in models after additional adjustment for myopia-related lifestyle risk factors. ORs were generated from logistic regression models, where myopia were treated as binary outcome measures. This method has been reported in previous publications [24, 25].

## Results

Two thousand four hundred thirty two grade 1 and 2346 grade 7 students were included in the analysis and their mean ages were 7.7 and 13.8 years for grade 1 and 7, respectively. There were more girls among grade 7 students compared with grade 1 students (48.3% vs. 44.8%). Comparison of myopia-related variables between the two samples indicated that grade 7 students had a greater body mass index (*P* < 0.001), had less myopic parents (*P* < 0.001), spent more time watching TV (*P* = 0.001) and playing computers (*P* < 0.001) and spent less time outdoors (*P* < 0.001) (Table 1).

**Table 1:** Characteristics of myopia-related variables among grade 7 and grade 1 samples

Variables	Grade 1 Sample	Grade 7 Sample	P
Girls(%)	1089 (44.8)	1133 (48.3)	0.015
BMI, kg/m2 (SD)	15.3 (2.5)	18.9 (2.7)	< 0.001
Parental myopia(%)	352 (14.5)	129 (5.6)	< 0.001
Time on reading and writing per day after school, hours (SD)	0.92 (0.57)	0.93 (0.67)	0.38
Time on computer per day, hours (SD)	1.42 (1.30)	0.90 (0.90)	< 0.001
Time outdoors per day, hours (SD)	2.60 (0.19)	1.34 (1.03)	< 0.001
Time on watching TV per day, hours (SD)	0.79 (0.43)	1.38 (0.91)	0.001

The distributions of cylindrical values, spherical values, SEs and ALs in the two samples are shown in Table 2. The crude mean SE was 0.87 D and − 0.27 D while the mean AL was 22.64 mm and 23.59 mm in grade 1 and grade 7 students, respectively. The distribution of SE was skewed towards more myopic values in both cohorts and neither SEs nor ALs were normally distributed (all P for Kolmogorov-Smirnov test < 0.001).

**Table 2:** Distributions of cylindrical value, spherical value, axial length and spherical equivalent refraction among grade 1 and 7 samples

	Mean	Median	Standard error	Standard deviation (Diopter)	Skewness	Kurtosis	Inter Quartile Range (Diopter)	Kolmogorov-Smirnov test
Grade 1 sample								
Cylindrical value (D)	−0.27	− 0.25	0.01	0.35	−3.91	25.51	0.25	< 0.001
Spherical value (D)	1.01	1.00	0.01	0.71	−0.17	30.87	0.50	< 0.001
Axial length (mm)	22.64	22.65	0.01	0.69	0.10	0.74	0.89	< 0.001
Spherical equivalent (D)	0.87	0.88	0.01	0.68	−1.37	40.46	0.50	< 0.001
Grade 7 sample								
Cylindrical value (D)	−0.36	−0.25	0.01	0.39	−5.88	66.64	0.25	< 0.001
Spherical value (D)	−0.09	0.25	0.03	1.43	−1.53	6.12	1.25	< 0.001
Axial length (mm)	23.59	23.51	0.02	0.90	0.52	0.80	1.12	< 0.001
Spherical equivalent (D)	−0.27	0.15	0.03	1.46	−1.69	6.27	1.38	< 0.001

*D* diopters

Table 3 compares the prevalence of myopia (SE < − 0.5D), high myopia (SE < − 5.0D) and mean ALs between the two samples. Grade 7 students had a higher prevalence of myopia (29.4% vs. 2.4%; *P* < 0.001) and high myopia (0.4% vs. 0.1%; *P* < 0.001) compared with grade 1 students. Grade 7 students also had longer ALs (23.50 mm vs. 23.37 mm; *p* = 0.004) after adjusting for the effect of gender, height and other myopia-related risk factors.

**Table 3:** Prevalence of myopia, high myopia, mean spherical equivalent and axial length in grade 1 and grade 7 samples

	Grade 1 Sample (*n* = 2432)	Grade 7 Sample (*n* = 2346)	P
Prevalence of myopia (SE < − 0.5 D) (%)			
Sex adjusted	2.4 (1.1–3.7)	29.4 (28.1–30.8)	< 0.001
Prevalence of high myopia (SE < −6.0 D) (%)			
Sex adjusted	0.1 (0.0–0.3)	0.4 (0.2–0.6)	< 0.001
Spherical equivalent (D)			
Sex adjusted	0.87 (0.82–0.91)	−0.27 (−0.32- -0.22)	< 0.001
Multivariate adjusted^a^	0.59 (0.37–0.80)	− 0.24 (− 0.31- -0.17)	< 0.001
Axial length (mm)			
Sex adjusted	22.64 (22.61–22.67)	23.60 (23.57–23.63)	< 0.001
Multivariate adjusted^a^	23.07 (22.94–23.20)	23.51 (23.47–23.55)	< 0.001

^a^Adjusted for sex, height, time for reading and writing after school, time outdoors, time on computers, time on watching TV and parental myopia

Finally, we estimated the reduction in odds of myopia associated with grade 7 students with adjustment of myopia-related factors. Adjustment for time spent on reading and writing after school per day led to a reduction in the excess prevalence of myopia in grade 7 students by 15.1%. In addition, adjustment for time outdoors reduced the excess prevalence of myopia in grade 7 students by 33.4%. Adjustment for other myopia-related variables did not significantly increase or reduce the excess prevalence of myopia in grade 7 students. (Table 4).

**Table 4:** Effect of potential explanatory factors on the excess prevalence of myopia in grade 7 sample compared with grade 1 sample

Model	Myopia(SE < −0.5 D)			
	OR	95% CI	P	% Reduction excess prevalence
1	18.22	13.74–24.16	< 0.001	Reference model
2	19.93	14.94–26.58	< 0.001	−9.9
3	17.96	13.51–23.88	< 0.001	15.1
4	12.47	7.39–21.05	< 0.001	33.4
5	19.09	14.27–25.55	0.012	−5.1
6	15.54	8.73–27.66	< 0.001	34.7

Model 1:sex; model 2:sex and parental myopia; model 3:sex and reading and writing per day after school; model 4: sex and time outdoors per day; model 5:sex and playing games or using computer; model 6:sex, parental myopia, reading and writing per day after school, time outdoors per day, playing games or using computer

*OR* odds ratio, *CI* confidence interval

## Discussion

In this school-based study of two samples with a difference in age of about 6 years, we reported a relatively lower prevalence of myopia in grade 1 and grade 7 students in rural China. We also found that approximately one-third of the excess prevalence of myopia in grade 7 students was explained by the reduction in time outdoors. Our study suggests that environmental factors such as time spent outdoors may play a major role in the development of school myopia in Chinese children.

High prevalence of myopia has been observed in many Chinese communities such as Shanghai [26], Guangzhou [27], Singapore [28], Hong Kong [29] and Taiwan [30]. Even in some rural areas in China, high prevalence of myopia was also reported among school-aged children [31, 32]. Thus, Chinese communities are regarded to have a prevailing concept of an “epidemic” of myopia [33]. However, it is still debatable whether the high prevalence is merely driven by environmental exposures associated with Chinese specific cultures. Our study found that myopes were very few among grade 1 students who have not been exposed to myopigenic factors such as spending large amounts of time on near work activities and less time outdoors. The prevalence of myopia among grade 1 students in our study is comparable to many other Asian communities which are characterized with low prevalence of myopia such as Cambodia [34], Laos [35] and Nepal [36]. Nowadays, most Chinese children in urban cities attend preschools such as kindergartens or childcare centers, and the syllabus in these settings is structured and vigorous, often being integrated with information technologies. Exposure to such a schooling system at an early age may result in an early onset of myopia. Even preschooling in urban China is highly competitive, academically oriented and emphasizes on very early educational achievements. The situation in our study site is different from most in other areas. For example, based on the survey results, most study participants have not attended preschools. Therefore, it is possible that our study participants were under less education ‘pressure’ than their counterparts in other areas, especially in urban cities. This may reflect a combination of lower level of reading exposures and nearwork loads, and corresponding higher levels of time outdoors. Although our study indicated that grade 7 students who have completed the primary school had a much higher prevalence of myopia compared with grade 1 students, the estimate was much lower compared with many reported in other studies of similar ages. Therefore, our findings emphasized the importance of the environmental impacts on school myopia.

Our study also revealed that there were little children with high myopia in both cohorts. This may be explained by the traditional view that genetic factors might have greater impact on high or extreme myopia while environmental factors may play a more important role in mild or moderate myopia. However, this argument is challenged given the recent rise in the prevalence of high myopia [26], which must have an environmental explanation. Further studies are need to distinguish between “old” high myopia, when educational pressures were low and is probably genetic, and “new” high myopia which has increased rapidly in younger cohorts, which is probably environmentally driven.

The implications of the findings in this study might be discussed. Although myopia prevalence was much higher among grade 7 students compared with grade 1 students, a 33% of the excess prevalence was due to reduction in time outdoors on the older cohort. We think that this is not sufficient to turn around the myopia epidemic in many of Asian communities, but the result suggests that general application of outdoor intervention during primary schooling might reduce the prevalence of myopia from over 80–90 to 50–60% in many Asian communities characterized with an extremely high prevalence of myopia. This reduction is clinically significant and may also reduce the burden of pathologic myopia morbidities. While the effect of outdoor intervention on the protection of school myopia has been confirmed [37], the cost-effectiveness of outdoor programs integrated within school curriculums to prevent myopia should be evaluated.

The strength of the study included a large sample size, standardized measurement of refractive error and the inclusion of two samples with different ages. There were some limitations of the study, which need to be acknowledged. First, the study is a school-based study rather than a population-based one. The prevalence estimates might be distorted as children who dropped out of schools were not included. However, school attendance rates of students in 2015 in Mojiang were about 99% due to the compulsory education system in China. Thus, we expected that the non-responder bias is minimal. Second, information on risk factors was collected by questionnaires, which might be potentially inaccurate due to recall biases. Finally, myopia-related risk factors included in this study could only explain about one-third of the excess prevalence of myopia in the older cohort and a large proportion of the excess prevalence might be captured by other factors which was not captured in this study.

## Conclusion

In conclusion, this study reported relatively lower prevalence estimates of myopia and high myopia in a Chinese population. Our study suggests that generation-specific environmental exposures may play a major role in the increasing prevalence of myopia between different generations. Further studies are needed to understand the detailed environmental and lifestyle changes which underline the risk of myopia between generations.

## Abbreviations

AL: Axial length
CI: Confidence interval
D: Diopters
OR: Odds ratio
SE: spherical equivalent
